# Experiencing dementia: How does assessment of cognition and language relate to daily life?

**DOI:** 10.1177/1471301220945832

**Published:** 2020-08-05

**Authors:** Sophia Lindeberg, Christina Samuelsson, Nicole Müller

**Affiliations:** Department of Biomedical and Clinical Sciences, 4566 Linköping University, Sweden; Department of Speech and Hearing Sciences, 8795 University College Cork, Ireland; Department of Biomedical and Clinical Sciences, 4566 Linköping University, Sweden

**Keywords:** dementia, clinical assessment, diagnosis, communication, interaction, daily life

## Abstract

This Swedish study investigates how persons living with dementia report their experiences of cognitive and linguistic testing, as well as their perspectives on the communicative resources and barriers they experience in daily interactions. Eight dyads were included in this qualitative exploratory study; eight persons with dementia and eight family members with whom they interact with daily. Semi-structured interviews, with questions focusing on experiences of diagnostic pathways as well as communicative and cognitive function in daily life, were carried out together with standard clinical testing. The data were analysed using qualitative content analysis. The results shed light on the experiences of uncertainty during the dementia assessment process related to the assessment tasks, the consequences of the assessment and receiving a diagnosis. We interpret this as a result of the unfamiliar clinical focus on function as measured in decontextualised tasks, compared to the participants’ view based on their abilities in everyday life. The study also reveals that adjustments in daily life that are necessitated by the consequences of neurological change are often developed in collaboration between the person with dementia and their conversation partners. There are, however, reports of conflicting feelings by the persons diagnosed with dementia, and by their families, as well as their views on how to best handle change, while maintaining a sense of being a competent person through the progression of disease.

## Introduction

Clinical guidelines for the provision of dementia services stress the importance of taking the priorities and individual needs of persons with suspected dementia into account throughout the diagnostic process and in service provision (Socialstyrelsen, 2017). Incorporating patient values and wishes is one of the three pillars of evidence-based practice, together with the best scientific evidence and clinical experience (Hauser, 2010). The delicate issues of balancing the standard clinical evaluation and taking into account the families’ wishes and needs are something with which the clinicians may struggle; for example Krohne et al. (2013) show that clinicians acknowledge a tension between ‘standardisation demands’, on the one hand, and individualisation, on the other hand (Krohne et al., 2013). A British study on dementia pathways shows that persons with suspected dementia may experience the assessment period as a generic process, rather than it being adapted to individuals (Manthorpe et al., 2013). The authors concluded that the experiences were closely related to how the healthcare practitioners communicate with patients. Furthermore, the lead up to assessments, once the decision has been taken to seek diagnostic evaluation, may entail a great deal of anxiety and uncertainty (Cahill et al., 2008). The experiences of the diagnostic assessment itself have also been shown to be demoralising, partly due to the unfamiliar setting. Assessment results can also be difficult to make sense of, and this may cause concern and worry (Keady & Gilliard, 2002; Samsi et al., 2014). Persons with suspected dementia and their relatives, likely, expect explanation of the symptoms (e.g. memory problems) they experience, as well as advice on how best to deal with them. However, a standardised diagnostic process (which prioritises the determination of a diagnostic category and severity) may not meet those expectations (Hill et al., 1995).

### Diagnostic pathways

In Sweden, where this study was conducted, 20,000–25,000 persons are diagnosed with dementia every year. Dementia diagnosis involves either the so-called basic dementia assessment (typically in primary healthcare settings) or an extended dementia assessment (in specialised dementia units, or memory clinics). Both assessment pathways involve neuroimaging, interviews and cognitive testing. The extended assessment is typically triggered when a basic assessment is non-conclusive (Socialstyrelsen, 2017).

### Cognitive and communicative function

In reviewing research on conversations and storytelling in dementia, Hydén (2018) concludes that progressive changes in dementia that affect conversational interaction include word-finding difficulties, increasing difficulties with references to the past, problems with managing conversational topics and frequently repeated statements. As has previously been reported, persons with dementia describe social activities as one of the most problematic areas in daily life (Aggarwal et al., 2003; Johansson et al., 2016; Small et al., 2000). However, it has been suggested that it might be the ‘loss on multiple levels – psychologically, socially and functionally’ that is perceived as the greatest difficulty when coming to terms with dementia (Robinson et al., 2011). Kitwood (1997) pointed out that there is a dynamic interplay between neurological deficits and social psychology, which is enacted in social, specifically conversational, interaction. During the course of dementia progression, new resources are also developed and used by persons with dementia, and their conversation partners, in order to manage cognitive-communicative change (Kindell et al., 2017). As Müller and Guendouzi (2005) have pointed out, compensatory communicative strategies may however vary in how productive they are, dependent on the interlocutors and the communicative context. Despite the growing body of research investigating clinical experiences, there is still a lack of research specifically addressing how persons with dementia make sense of the cognitive and linguistic assessment, as well as how these experiences are made sense of in light of change in daily life. Lindeberg et al. (2019) investigated the perspectives of healthcare professionals on diagnostic testing and disclosure. Their results indicate potential discrepancies between test results, patients’ and relatives’ descriptions of abilities in daily life, and informal clinical observations. Furthermore, the informal and formal information obtained during the diagnostic process may be weighed differently when deciding on a diagnosis, dependent on the clinical setting (Lindeberg et al., 2019).

## Aim

The study aims to gain insight into how persons with dementia, and family members, make sense of the dementia assessment process, as well as how they make sense of encounters with health care. The study also aims to explore how the experiences with clinical testing relate to perceived change of functioning in the context of everyday life, and potential cognitive-communicative change.

## Method

### Research design

The chosen qualitative exploratory design employed semi-structured interviews to best capture the participants’ own perspectives on clinical encounters and assessment and their experiences of communicative and cognitive function and interaction in daily life. This study was approved by the regional Ethics Committee in February 2016 (dnr 2015/348-31, 2016/487-32). All participants signed written consent before participating.

### Participants

Eight dyads took part in this study; each dyad consisted of a person with a diagnosis of dementia of any type and a family member with whom they interacted on a daily basis. Participants with a dementia severity that would have raised doubts about their ability to give informed consent were excluded from the study. The participants had been diagnosed with various types of dementia and had been assessed either within primary health care or through a memory clinic (see Table 1 for further participant characteristics). One of the participants is categorised under ‘dementia unspecified/primary progressive aphasia’ (PPA) since symptoms of progressive aphasia were noted during diagnostic assessment even though the preliminary diagnosis was ‘dementia (unspecified)’.

**Table 1.** Participant characteristics.

Participant with dementia	Diagnosis (no)	Assessment process	Family member
Age (mean)	Lewy body dementia (2)	Dementia assessment unit (no)	Daughter (2)
70–86 (78)	PPA (2)	Memory clinic (5)	Son (1)
Sex (no)	Mixed dementia (2)	Primary health care (3)	Spouse (5)
F (2)	Alzheimer disease (1)		
M (6)	Dementia unspecified/PPA (1)	Years since diagnosis 1–4	

PPA: primary progressive aphasia.

### Data collection

#### Overall set-up

The persons with dementia chose their own family member, and together with the first author (SL), the participants set the times and location for the interviews and assessments. The meetings included (1) a dyadic interview with both participants, (2) an interview with the participant with dementia alone, (3) testing with the participant with dementia, either in a single session or spread over two sessions: a test battery with clinical cognitive and language-based tests with (4) interviews between and after tests in order to capture experiences and perceptions during and after testing. All data collection, including interviews and testing, was carried out by SL, who has a background as a practicing speech-language pathologist working with acquired neurological disorders. All interviews and testing were audio recorded, as well as video recorded for those participants who agreed to this (*n* = 6). An interview guide (see Appendix 2) for the initial interviews was constructed, including questions in the areas of experiences of clinical evaluation and testing, dementia diagnosis, daily living, communicative and cognitive function, factors affecting communication and ways of overcoming possible barriers in interaction. The participants were also encouraged to raise their own topics of interest. The interview guide therefore served as a support during the single and dyadic interviews; however, the content of interviews varied, depending on the events and experiences that participants wished to share. One participant only agreed to be interviewed but did not wish to do the testing. This was also the first interview and was therefore set up as a pilot interview. However, since the protocol was not changed after the first interview, the data were included in the overall analysis.

#### Test battery

Testing was carried out with unstructured follow-up interviews after each test was completed. Time required for testing was adjusted according to the needs of the participants, and participants could take breaks between tests if they wished. The family members were not present during the test sessions.

The test battery (see Table 2) consisted of A-ning (Werner & Lindström, 1995), the Swedish version of the Boston Naming Test (BNT; Tallberg, 2005), FAS, animal and verb (Tallberg et al., 2008) and the Swedish version of the cognitive screening test Mini-Mental State Examination (MMSE-SR; Palmqvist et al., 2012). There are currently no dementia-specific language tests that are routinely used in Sweden. Therefore, A-ning and BNT were used because they are the most commonly used tests in the assessment of adult acquired language disorder (Blom Johansson et al., 2011) whereas BNT, A-ning, FAS, animal and verb are used in the assessment of language in dementia in some Speech Language Pathology (SLP) clinics in Sweden (Tallberg, 2012). The MMSE-SR is a widely used test (Ismail et al., 2010) that is also prioritised for dementia assessment, according to the Swedish National Board of Health and Welfare (Socialstyrelsen, 2017). The BNT and MMSE-SR were administered to all participants. For the remaining tests, however, adjustments in the number of tests carried out were made for each participant, depending on how effortful and time consuming the tests were for individual participants. With one participant with non-fluent PPA, the short version of the BNT was carried out. The results of MMSE-SR and BNT are presented in Table 1, together with the participant characteristics. None of the participants were given, nor did they ask for, their test score. The first interviews and testing with interviews were performed within 4 weeks for each participant.

**Table 2.** Test battery.

Ability assessed	Assessment tool	Test score for participants in the present study
Cognitive screening	MMSE-SR (Folstein et al., 1975; Palmqvist et al., 2012)	MMSE-SR (<30): 11–30
Orientation		
Immediate recollection		
Attention		
Delayed recollection		
Language tasks		
Figure copying		
Listening comprehension	A-ning (Neurolingvistisk afasiundersökning, *Neurolinguistic Aphasia Examination*) (Werner & Lindström, 1995)	
Informal spoken language		
Naming ability	Boston Naming Test (Kaplan et al., 1983; Tallberg, 2005)	BNT (*n* = 6, score < 60): 43–58
		BNT short version (*n* = 1, score < 15): 4
	Letter (FAS) and semantic category naming (animal and verb) (Tallberg et al., 2008)	
	Maximum naming within 60 seconds per letter/category	

MMSE-SR = Mini-Mental State Examination, Swedish Revised Version.

### Data analysis

The transcripts were analysed using qualitative content analysis, following Graneheim and Lundman (2004). Content analysis is a research technique suitable for categorising and making inferences from different types of data, for example transcripts from interviews (Krippendorff, 2019). The recordings were first transcribed verbatim by the first author, including non-verbal interaction when necessary to understand what the participant was referring to, or if non-verbal expressions were used instead of verbal expressions, for those interviews that also included video uptake. Each transcript was read through thoroughly several times by all authors, in order to gain familiarity with the data. After excluding sections that did not include relevant, codable content (for example when talking about preparing coffee) the data were uploaded to NVivo (version 12) and coded. Codable content was defined as relating to aspects of daily life, for example activities, social contacts or concerns related to their diagnosis, as well as previous or current contacts with health care. Transcripts were divided into meaning units, and each meaning unit in turn transformed into a condensed meaning unit. The condensed meaning units were translated into English, and English was used for subsequent coding and derivation of subcategories and categories (see Appendix 1 for examples of coding and categorisations). Throughout the analysis process, the authors discussed coding and categorising, and codes and categories were adjusted and agreed upon by consensus.

## Results

Two themes were generated: *Attempting to reconcile lived experiences with diagnostic testing and labels* and *managing tensions, developing resources, adjusting roles, actions and interactions* (see Table 3 for an overview of themes, categories and subcategories).

**Table 3.** Themes, categories and subcategories.

Theme	Category	Subcategory
Attempting to reconcile lived experiences with diagnostic testing and labels	Noticing change and initiating healthcare contact	Changes leading to initiation of healthcare contacts
		Initiating contact with health care
	Processing own and other’s views of function and dementia labelling	Reactions to diagnostic disclosure
		Being positioned by healthcare contacts, including dementia as a label
	Making sense of the assessment	Making sense of the assessment and its consequences
		Aspects affecting test results
Managing tensions, developing resources, adjusting roles, actions and interactions	Resources and barriers in conversation	Changes in function
		Experiences of tempo in interaction
		Conversational partners as a barrier of resource
		Settings as a barrier or resource
	Handling cognitive and communicative change	Collaboration and embodiment in conversation
		Using mnemonics and artefacts
		Avoidance
	The importance of activities and social participation	Continuing with earlier activities
		Sharing common ground in dementia groups
		Keeping up with earlier contacts

### Attempting to reconcile lived experiences with diagnostic testing and labels

The process of being diagnosed started with the participants *noticing change and initiating healthcare contact*. ‘Trigger experiences’ for contacting health care occurred when the person noticed changes in one or multiple abilities; these included physical abilities (e.g. walking difficulties, experiences of dizziness and having headaches) and cognition and communication. A son described his recollection of how getting lost while driving led his mother, who was later diagnosed with Alzheimer’s disease, to contact health care:

You might be out driving and got lost on your way to our place, and issues like that would happen once in a while. You start to wonder what’s going on, if you’re just stressed or what, and got lost and stuff like that, but… But then I think it was actually you who initiated that contact, I think you handled most of the contacts with the doctor in the beginning. (son, dyad 4)

Dyad 7 experienced changes in conversations relating to the husband becoming more and more quiet. This change led to them setting up an appointment with their local General Practitioner (GP). The GP interpreted the symptoms as due to depression, and it was not until later that the GP referred him to the memory clinic and he was diagnosed with PPA. During the diagnostic process, the participants started *processing their own and others’ views of function and dementia labelling.* During the interviews, both the participants with dementia and the family members most commonly used terms such as ‘memory loss’, or ‘memory changes’, rather than referring to diagnostic labels such as ‘Alzheimer’s disease’. However, dyads 1, 7 and 8, who were living with PPA, used the term ‘aphasia’. Dyad 1 explicitly distanced themselves from the label ‘dementia’, and evidently equated ‘dementia’ with memory impairment:

She doesn’t have dementia, it’s just difficulties in- she’s on top of things, it’s very upsetting if you’re treated like that. But I do think, when I’ve been there, it has improved, and this time it was even better. Somehow, they’ve understood that it’s not dementia in any way. […] Because there’s nothing wrong with her memory. (daughter, dyad 1)

Participants reported different reactions to diagnostic disclosure, ranging from the diagnosis being expected to ‘not knowing how much it would affect life’ (person with dementia, dyad 3). The same participant expressed, during both the individual and dyadic interviews, the hope that he would be able to learn how to drive again as well as a wish for a clinical breakthrough or an inclusion in a clinical trial, in order to be able to recover:

Anything, as long as it gives me hope […] and helps me get back to how it once was. (person diagnosed with Lewy body dementia, dyad 3)

One of the family members (dyad 10) was critical of the way in which the geriatrician had downplayed the effect of the symptoms of her husband’s aphasia, where the doctor had referred to him being ‘a big and strong man’ and that he would ‘get through this’. She experienced her husband as being deeply emotionally affected by the aphasia and explained how this comment was less than helpful. Nonetheless, overall the participants characterised their encounters with the health care as positive.

Additionally, the participants reported different experiences in relation to *making sense of the assessment* that took place during the clinical pathway. Several of the participants recalled efforts in trying to make sense of the tasks they were asked to perform and the reasons behind them, as seen in the quote below, from the initial individual interview:

They came in with some tray, right, and started asking about what that is. And there’s a piece of sugar there, right, and crap stuff like that they’re talking about. I’m looking for something more and walk out from there thinking ‘what the heck did I do there?’ But the nurses there, they don’t get what the issues are, and how you solve problems. [..] You don’t get any sense of that being anything they might get anything from… (person diagnosed with mixed dementia, dyad 5)

Furthermore, participants reported difficulties understanding how doctors drew conclusions from test results. A participant, who had been diagnosed with dementia four years previously, described the clinical encounter in which he had received the diagnosis, and how the test scores led to him losing his driving license:

And then [the doctor] pointed at some results of the testing, and I didn’t know what those scores meant. ‘Look here, you can’t drive a car’ – but which quality was missing, I don’t know. (person diagnosed with Lewy body dementia, dyad 3)

Despite his difficulties in making sense of the doctor’s conclusion, he did not raise this issue, rather ‘just took it’.

Participants also emphasised the importance of the relationship with the clinician in relation to test performance, where one participant described performing significantly better during a second testing session due to the overall encounter being more positive.

During and after the testing carried out as a part of this study, participants commented on the connections they perceived between tasks and scores. For example one participant explained high scores on the BNT by the test being easier for elderly persons than for younger persons due to the types of target words. Conversely, and more commonly, difficulties with specific tasks were explained as the task being dependent on the person’s background (age, personal experiences, education, etc.), such as a specific fruit not being common during one’s upbringing, or target words were judged to be uncommon in daily life. The participant in dyad 3 (P3 in excerpt below), diagnosed with Lewy body dementia, contrasted everyday activities with performing a listening task from the neurolinguistic aphasia assessment A-ning:

SL: What did you think about that task?

P3: Well, it´s (confusing) enough, you feel (embarrassed).

SL: You feel embarrassed?

P3: Yes, uncertain. It might be because you’re not used to listening in this way. I read my newspapers, and…

For a dyad living with non-fluent PPA, language changes had severely impacted conversation and participation in various activities. In contrast, the diagnostic assessments, as well as the testing carried out as part of this study, were experienced as easy in comparison with casual conversation. This mismatch in performances made the dyad feel that their difficulties did not come to light during testing.

### Managing tensions, developing resources, adjusting roles, actions and interactions

All participants spoke about changes they had to adjust to as a consequence of dementia. These included changes in cognitive-communicative ability, as well as sleep disorders, having a hard time recognising faces and hallucinations. Regarding *resources and barriers in conversation*, communication with others was affected by barriers such as word-finding difficulties, slower speech, slurred speech (for the participants diagnosed with Lewy body dementia), difficulties entering into conversations, repeated statements (as described by family members) and fatigue after long conversations. These difficulties would also result in misunderstandings, causing irritation between spouses. Group size, as well as how well you know your conversation partner, was seen as either a barrier or resource, where familiarity and a smaller group size would have a positive effect on conversation. Several of the participants also mentioned that it was harder to speak on the phone than face-to-face. Fluctuation in cognitive-communicative function, over the course of a day or between days, was also a prominent factor.

As mentioned, several dyads brought up difficulties for the person with dementia entering conversations. One of the participants, diagnosed with Lewy body dementia, was, however, clear about not wanting his conversational partners to slow down the tempo, not wanting them to have to adjust to him.

Three of the participants took part in weekly group meetings for persons with dementia in an activity centre, where casual conversations were the focal activity. These participants emphasised their appreciation of the group leaders and their role as communication facilitators, contributing to the good atmosphere in the group:

The two girls try to keep the discussion going, among us ‘mortals’ and it’s quite nice. […] And the girls, the nurses, are quite good at bringing out- asking questions and getting us to talk. I guess that’s their task, really. (person with Lewy body dementia, dyad 2)

A daughter–father dyad talked about the social gatherings that took place in the father’s residential home which shed light about their views on conversation abilities in dementia. The father described how for him, the degree of cognitive decline of the person he was placed next to during mealtimes or other activities largely determined whether he enjoyed them. The daughter specifically stated that staff should pair residents off according to ability in order to foster interaction.

In addition, a few of the participants brought up the issue of talking over the head of, or speaking for, the person with dementia. This was a concern most often raised by the spouses of the person with dementia, related to their own communicative pattern. One of the spouses described how she tries to actively give the floor to her husband, who was diagnosed with PPA, but that in contrast, their son calls her the husband’s ‘spokesperson’ (*sw.* språkrör). However, when the husband was, during the individual interview, asked about his feelings of her speaking for him, he described that as a ‘good’ thing. Another participant mentioned that having the family member close by gave him a sense of security. One of the dyads raised the tension between wanting to be helpful, and being perceived as talking on behalf of the person with dementia, from their son’s perspective:

‘Be quiet’, [the son] says. ‘Let dad $finish$ talking, right?’ That´s what he says. [turns towards her husband:] Because you just want to help out. I can hear, I know how your thoughts go, and what you think about and what situations you are talking about. (wife of person with Lewy body dementia, dyad 3)

One woman with dementia spoke about feeling excluded from conversations during meetings at her memory clinic, describing how no one would talk to her when there were more participants in the room, and how she would ‘feel excluded’.

Being able to communicate well in clinical encounters was perceived as highly dependent of the clinician, and the relationship with the healthcare professional was seen as important. Four of the participants had ongoing SLP contacts, and two of the participants with PPA specifically brought up an experience of conversations being more fluent with their SLP. This was partly due to ‘liking’ the SLP, indicating the importance of the relationship. One participant’s SLP also used picture support in conversation, which the participant considered helpful.

The participants had different ways of *handling cognitive and communicative change*. However, a common strategy was collaboration in order to overcome communication barriers. One of the participants with dementia would ask his wife a set of questions in order to prompt himself:

One can try by saying ‘can you remember what was in this or that place?’ Or by saying ‘have you heard about this or that on TV?’ And then I might remember what it was. (person with mixed dementia, dyad 5)

Mnemonics and artefacts were also used, but in some dyads, their use was restricted to specific settings. As mentioned, one dyad explained how pictures were used during SLP encounters. When asked if they also use pictures or any other artefacts at home, the wife described their way of handling change collaboratively:

By and large, it works for us. But really, it is very quiet. But it’s not like there’s anything that’s unclear. Because if [the husband] says something that one doesn’t quite understand, I’ll just ask again. So it’s not like you don’t understand what I’m saying and I don’t understand what he’s saying. (wife of man diagnosed with Primary Progressive Aphasia, dyad 7)

Dyad 8, however, reported that conversations were highly problematic but did not mention any compensatory strategies, such as writing. Furthermore, there were occasions where the wife had difficulties in interpreting her husband’s attempts at making himself understood:

You often do this [holds up two fingers and turns them]. But nobody gets it. Using the fingers. (wife of man diagnosed with Primary Progressive Aphasia, dyad 8)

During the interviews, the husband in dyad 8 supported some of his utterances with writing, for example when mentioning a specific year that was hard to verbally express, and sometimes to prompt himself by tapping on the table.

Artefacts and locations were also used strategically. For instance, one man, who lived on his own, structured his room and papers in a certain way in order to help himself remember appointments. If he had an appointment at the doctor’s, for example he would put the appointment letter in a specific place on his living room table, where he knew he would see it often. He used this location solely for the purpose of constructing tangible reminders. Receiving the appointments in writing was therefore important for him, in order to maintain this strategy. Calendars were also important, most often as described by the family members during the dyadic interviews. One woman with dementia, who lived on her own, used the calendar to keep track of appointments; however, her son indicated that the calendar might no longer be effective, and that some other reminders really would be needed.

There were also reports of avoiding certain activities that were dependent on, or included, conversations with other persons. For example dyads described becoming silent with each other in order to avoid misunderstandings. Reduced conversational initiative by the person diagnosed with dementia was an additional cause. One of the couples explained how aphasia led to the participant avoiding taking daily walks, in order to avoid seeing neighbours who would want to converse. Taking long walks used to be the couples’ main way of keeping physically active. Instead, they tended to take long walks mainly when abroad.

Participants emphasised *the importance of activities and social participation* and illustrated this by talking about activities that they maintained, despite the decline and challenges they experienced. Many of the participants were socially active and kept close contact with old friends and colleagues. Two of the participants did sports together with former colleagues on a regularly basis, and maintaining long-established contacts was seen as very important. Positive aspects included being familiar with each other and having a certain ‘jargon’. New acquaintances were also made in the dementia activity groups, where ‘non-pretentious’ conversations could take place. One dyad reported that they would attend different activities organised by the local dementia centre almost on a daily basis. Despite this, the husband (with dementia) stated, during the individual interview, that he felt lonely and ‘never sees anyone’. This could therefore reflect the importance of also staying in contact with older acquaintances from before the diagnosis.

## Discussion

Eight persons diagnosed with dementia, as well as eight family members with whom they interacted with on a daily basis, took part in this study exploring cognitive and communicative ability from a perspective of clinical experiences and everyday life with dementia. Participants find it difficult to make sense of the assessment of cognition and language; a chief concern is function in daily life, as well as the impact any changes may have on daily activities. The clinical diagnostic testing is, however, experienced as decontextualised, focusing on abilities that may be difficult to relate to everyday life. Additionally, experiences of cognitive and communicative change in daily life have a social facet that affects the way in which the person with dementia experiences and adjusts to change in daily interaction.

### Evaluating function

The results shed light on the tension between decontextualised evaluative methods, and the contexts in which the participants make sense of everyday function. Tasks carried out in the clinical setting often do not directly relate to tasks carried out in daily life, such as target words in naming tasks containing words uncommonly used in everyday life. Participants’ reactions to and reflections on the tests show that they, however, attempt to relate them to abilities in daily life. This can only meet with limited success since norm-referenced tests of cognition and language are designed to be context independent (in as far as this is possible). Clinicians need to be aware that patients are likely to want to make sense of the testing experience in terms of what they are familiar with, that is their own skills and difficulties in their daily lives.

In line with our results, Saunders et al. (2011) describe how patients with cognitive impairment going through cognitive testing might explain a lack of knowledge as differences in lived experiences. Patients may also have a hard time following what the tests assess (Krohne et al., 2011), as well as grasping the professionals’ explanations of test results (Samsi et al., 2014). The relation between the test results and their consequences can also be hard to make sense of when not seen as applicable to the target task itself. One example is losing one’s driving license, based on cognitive test performances rather than driving itself. Research shows that clinicians carrying out testing are often aware of patients’ difficulties in understanding the meaning of test scores, resulting in clinicians either choosing not to give feedback on the test scores, or contextualising the scores (Krohne, 2014). On the basis of our results, we would however argue that omitting information about the use of test scores as a part of the assessment may not be beneficial for the patients’ sense-making either since patients do try to make sense of how the scores obtained from testing are interpreted by the clinician.

While the clinical setting has been described as problematic due to its unfamiliarity (Cahill et al., 2008; Keady & Gilliard, 2002; Manthorpe et al., 2013), the participants in this study did not indicate that the setting affected the experience. Rather, the content and structure of the tests and how clinicians use the results seemed to be the main causes of uncertainty. Interestingly, however, one of the participants experienced a difference in test performance depending on which clinician carried out the test, indicating the importance of the relationship between the clinical professional and patient.

Furthermore, despite difficulties in grasping the meaning, and use of, certain parts of the diagnostic process, patients may not signal any problems, as was seen in this study. This is important since any latent difficulties in making sense of the process may lead to miscommunications between the clinical professional and care recipient, as well as difficulties in making sense of the diagnostic pathways.

This study also sheds light on how people understand the diagnostic label dementia, namely that memory impairment is considered a defining characteristic of the condition. Persons with PPA may not understand why they are being evaluated in a memory clinic if they do not experience memory difficulties, and they understand dementia as primarily involving memory loss (which is reinforced by the term ‘memory clinic’, incidentally). Preconceptions are also seen in the way which persons diagnosed with dementia, as well as their family members, describe communication in dementia, where there seems to be a tendency to view communication with persons with dementia as less meaningful. Ultimately, these preconceptions may lead to situations where potentially meaningful conversations are avoided.

### Adjusting to change

Kitwood (1997, p. 51) described a ‘dynamic interplay between neurological impairment and malignant social psychology’, linking social consequences to change as a result of neurological decline. We draw upon three examples that display the complex ways in which cognitive and communicative consequences of dementia, on the one hand, and social consequences, on the other, are experienced in daily interaction:

1. In line with a previous study by Small et al. (2000), our results show how persons with dementia experience conversations as too fast. However, our results show that what may be seen as helpful adjustments, such as conversation partners ‘slowing down’ in conversation, may not always be preferred when persons with dementia perceive that others have to adjust their conversation to accommodate them. Steeman et al. (2007) found that rather than cognitive decline being the main source of concern, being ‘someone of value’ was. Thus, being someone to whom others need to adjust becomes a larger concern than not keeping up in conversation. This aspect may be due to three interrelated issues: (i) adjustments made by others in conversations may affect the way in which the person with dementia views him- or herself (including self-esteem), (ii) how he/she is viewed by others (including communicative competence), as well as (iii) not wanting to impose on others. This study also sheds light on avoidance behaviours, such as avoiding taking walks due to a fear of meeting neighbours and having to converse with them. This illustrates that a self-image as an incompetent communicator can have potentially far-reaching consequences for one’s physical and mental health.
2. Hamilton (2019) points out that triadic conversations including an individual with dementia ‘opens up the possibility that one or another of these participants will be spoken *about* or *for* in their presence’ (Hamilton, 2019, p. 76). In a case study, Purves (2009) showed how a woman with non-fluent PPA and her interlocutors described ‘speaking for another’ as interactionally challenging, but, at other times, as a resource in conversation. The analysis of the participants’ interaction revealed different collaborative patterns regarding, for example how involved both partners were with producing the narration, as well as the amount of co-authoring that was going on (Purves, 2009). In the present study, speaking for someone is mainly described as problematic by the family members, but not the person with dementia, indicating a difference in how the participation is viewed. Several of the persons with dementia describe how they, when having word-finding issues, give cues to their spouse, in order to help out with finding a word, or an utterance. Viewing the person with dementia as an active participant in *giving* the floor, as opposed to the conversation partner *taking* the floor, is also supported by other interactional research demonstrating how persons with dementia are active in co-constructing utterances, by initiating the spouse to take over the floor (Nilsson et al., 2018). Nonetheless, being talked ‘over the head’ (dyad 1) of in clinical encounters was negatively perceived, resulting in a feeling of being ‘excluded’. Talking about someone when they are present is a type of ignoring, part of malignant social psychology (Kitwood, 1997). Österholm and Samuelsson (2015) have demonstrated how persons with dementia are ignored, either through talking over their head or by not responding to the persons’ initiatives, during assessment meetings.
3. The perceived ability to take part in a conversation is not merely a result of decontextualised linguistic and cognitive abilities. This can be exemplified by the man with steadily declining language function (dyad 7), who describes how he used to speak less due to being embarrassed about the aphasia, compared to later, after coming to terms with this condition.

There are some methodological considerations that we wish to discuss. Firstly, the ways of making sense of tests that are performed in the homes of a participant with established dementia will not fully depict the feelings that are experienced during actual diagnostic testing. Additionally, the tests that are carried out as a part of this study may not have been the tests that the participants performed as part of their dementia assessments. Nonetheless, this study sheds light on the ways in which patients may try to make sense of the assessment process and the nature of the tasks involved. Furthermore, carrying out testing outside a formal procedure, offered means to explore sense-making during the assessment, after each task was carried out. To our knowledge, this is the first study exploring the experiences of persons with dementia, by carrying out interviews between tasks. In order to capture the process of sense-making related to dementia pathways, it was fruitful to also interview the patients about their clinical experience. In this study, the participants were interviewed between one and four years after the dementia assessments. While this delay may result in memories being more distant or re-formed along the way, we argue that this offers an important addition when exploring the long-term effects of coming to term with the assessments and its consequences. The small sample of participants may be seen as a limitation in this study. Further explorations of the experiences in the different diagnostic pathways are therefore needed.

## Conclusions

This exploratory study shows how participants may find it difficult to link experiences of clinical testing with experiences of dementia in daily life. This is perhaps not surprising since clinical diagnostic testing is decontextualized by design, whereas sense-making is done in terms of lived experience of deficits and skills. However, since the participants’ chief concern is how dementia will impact their daily lives, it is not surprising that they seek to make sense of clinical testing in light of their lived experiences and their own perceptions of skills and deficits. Therefore, understanding the process and outcomes of the assessment process can be of importance for patients’ future sense-making as part of coming to terms with the evaluation process and with diagnostic disclosure. As a result, clinical professionals need to acknowledge, and consider, patients’ perspectives of cognitive and communicative ability in daily life, as well as acknowledge that it can be hard for patients to make sense of the clinical conclusions drawn from the clinical testing. After all, and as our results support, families initiate healthcare contact based on concerns in everyday life. Some participants, however, feel that their concerns are not properly addressed.

Related to experiences in everyday life, this study highlights the varied ways in which families living with dementia address cognitive and communicative change collaboratively, and how the experience of dementia is primarily a social one, where participants need to make adjustments and learn to live with dementia within their own social and interactional frameworks. Our results show that in the course of dementia progression, changes in cognitive and communicative function do affect interactional patterns. However, changes in interaction are also a result of adjustments made by the persons with dementia and their interactional partners. The social consequences are also closely related to reactions to the diagnosis obtained, as well as self-image and a sense of communicative competence as an interactional partner. As a result, clinicians need to take into account families’ joint experiences of change in everyday life and also include interactional partner’s when giving advice.

## Appendix 1. An example of the coding process.

**Table:**

Meaning unit	Condensed meaning unit	Code	Subcategory	Category	Theme
‘And you’re used to a certain pitch. Now when it goes down, a bit thick. It bothers me. You’re used to lecturing all day, and managing. So it’s bad’.	Lower pitch and thick voice	Voice deterioration	Changes in function	Resources and barriers in conversation	Managing tensions, developing resources, adjusting roles, actions and interactions

## Appendix 2. Interview guide.

**Table:**

When the dementia assessment was carried out and who initiated it
Experiences of dementia evaluations
Tests carried out
Strengths and weaknesses that appeared and how they correspond with own experiences of function
Communication in daily life (resources and barriers)
Situations where the communication works well or not well
Barriers in cognition and language/resources/strengths
Strategies to overcome barriers
What should be noted and evaluated in healthcare settings
Thoughts concerning length, setting and layout of assessments
Thoughts about involving family, and if so – how

## Appendix 3. Transcription conventions.

**Table:**

-	An abruptly ended or cut off word
[…]	Omitted words or sentences
$	Smiley voice
CAPS	Word stronger than surrounding words
[]	Transcribers’ description
()	Uncertain transcription
happens	Stressed word
